# Cognitive dysfunction improves in systemic lupus erythematosus: Results of a 10 years prospective study

**DOI:** 10.1371/journal.pone.0196103

**Published:** 2018-05-03

**Authors:** Fulvia Ceccarelli, Carlo Perricone, Carmelo Pirone, Laura Massaro, Cristiano Alessandri, Concetta Mina, Massimo Marianetti, Francesca Romana Spinelli, Guido Valesini, Fabrizio Conti

**Affiliations:** 1 Lupus Clinic, Dipartimento di Medicina Interna e Specialità Mediche, Sapienza University of Rome, Viale del Policlinico, Rome, Italy; 2 Dipartimento di Neurologia e ORL, Sapienza Università di Roma, Rome, Italy; Peking University First Hospital, CHINA

## Abstract

**Objective:**

Cognitive impairment (CI) has been described in 3–80% of Systemic lupus erythematosus (SLE) patients but only short-term studies evaluated its over-time changes, suggesting that CI is usually a stable finding. We aimed at evaluating the changes of SLE-related CI in a 10-years prospective single center cohort study.

**Methods:**

We evaluated 43 patients (M/F 5/38; mean age = 45.7±10.1 years; mean disease duration = 230.8±74.3 months) at baseline (T0) and after 10 years (T1). A test battery designed to detect fronto-subcortical dysfunction across five domains (memory, attention, abstract reasoning, executive and visuospatial function) was administered. A global cognitive dysfunction score (GCD) was obtained and associated with clinical and laboratory features.

**Results:**

Prevalence of CI was 20.9% at T0 and 13.9% at T1 (P = NS). This impairment was prevalently mild at T0 (55.5%) and mild or moderate at T1 (36.3% for both degrees). After 10 years, CI improved in 50% of patients, while 10% worsened. Impaired memory (P = 0.02), executive functions (P = 0.02) and abstract reasoning (P = 0.03) were associated with dyslipidemia at T0. Worsening of visuospatial functions was significantly associated with dyslipidemia and Lupus Anticoagulant (P = 0.04 for both parameters). Finally, GCD significantly correlated with chronic damage measured by SLICC/damage index at T0 (r = 0.3; P = 0.04) and T1 (r = 0.3; P = 0.03).

**Conclusions:**

For the first time, we assessed CI changes over 10-years in SLE. CI improved in the majority of the patients. Furthermore, we observed an improvement of the overall cognitive functions. These results could suggest that an appropriate management of the disease during the follow-up could be able to control SLE-related CI.

## Introduction

Systemic Lupus Erythematosus (SLE) is an autoimmune disease characterized by a multifactorial etiology, in which genetic and environmental factors determine disease development [1]. The production of several autoantibodies, often associated with particular phenotypes, characterizes the disease. [2, 3]

Among the different clinical manifestations, neuropsychiatric involvement can affect up to 90% of SLE patients.[4–9] A wide heterogeneity of neurological and psychiatric manifestations characterizes the neuropsychiatric SLE (NPSLE), as demonstrated by the 19 SLE-associated neuropsychiatric syndromes included in the standard nomenclature proposed by the American College of Rheumatology (ACR). ] Indeed, NPSLE could widely vary with reference to severity, ranging from headaches non responsive to narcotics to life-threatening conditions. Sometimes the diagnosis could be very difficult due to the absence of specific biomarkers or imaging tools, able to discriminate SLE-related manifestations from other conditions, such as infections or drug-related adverse events. [10, 11]

Cognitive impairment (CI) represents one of the most common neuropsychiatric feature in SLE patients, with a prevalence ranging from 3% to 80%. [12–15] This wide range could depend from several reasons, such as different population assessed, neurocognitive tests applied to evaluate the manifestation and absence of adequate control groups. Moreover, it should be considered that some studies evaluated only symptomatic patients while other studies applied a universal assessment. [5–9, 15]

From a pathogenic point of view, NPSLE development has been related to the presence of autoantibodies and cytokine-mediated neuronal dysfunctions, vasculopathy, and coagulopathy.[16] Several autoantibodies, potentially exerting a pathogenic role, have been associated with this involvement: among these anti-phospholipids (aPL), anti-endothelial, anti-P ribosomal proteins, human N-methyl-D-aspartate (NMDA) receptor types NR2a or NR2b (anti-NR2) antibodies, anti-neuronal, anti-GAPDH. [17–21] In particular, SLE-CI seem to be caused by a damage localized in the fronto-subcortical circuits, as demonstrated by the involvement of domains related to executive functions, attention, learning and recall, verbal and nonverbal fluency, language, visuospatial skills, and motor dexterity. [7–9]

With regard to the assessment of SLE-related CI, the ACR Ad Hoc Committee on Neuropsychiatric Lupus nomenclature proposed in 1999 a brief research battery able to quantify these dysfunctions. [4]

So far, the studies assessing cognitive impairment in SLE patients are mostly cross‐sectional, without providing information about over-time changes.

To the best of our knowledge, only four longitudinal studies have been conducted, with a maximum follow-up of 5 years. [22–25] Taken together, these studies suggested that CI is a relatively consistent and stable finding in SLE patients. [22–25] Nonetheless, longer follow-up may better depict the evolvement of this neuropsychiatric manifestation.

Thus, in the present 10-year prospective study, we aimed at evaluating the changes of CI in a single center SLE cohort. Secondly, we evaluated the correlations between CI and clinical and laboratory SLE-related features.

## Patients and methods

Fifty-eight adult patients affected by SLE according to the ACR revised criteria, were enrolled consecutively in this longitudinal study at the Lupus Clinic, Sapienza University of Rome. [26]

Written informed consent was obtained from each patient and the local ethic committee approved the study design. The baseline features of this cohort were described in a previous study. [15]

According to the study protocol, the patients were evaluated at baseline (T0) and after 10 years (T1).

### Clinical and laboratory evaluation

Study protocol included complete physical examination and blood drawing. The clinical and laboratory data were collected in a standardized computerized electronically filled form including demographics, past medical history with date of diagnosis, co-morbidities, and previous and concomitant treatments.

Each subject underwent peripheral blood sample collection. The study protocol included the determination of autoantibodies and the evaluation of C3 and C4 serum levels. Specifically, ANA has been determined by means of indirect immunofluorescence (IIF) on HEp-2 (titer ≥1: 160 or ++ on a scale from + to ++++), anti-dsDNA with IIF on *Crithidia Luciliae* (titer ≥1: 10), ENA (including anti-Ro/SSA, anti-La/SSB, anti-Sm, and anti-RNP) by ELISA assay considering titers above the cut-off of the reference laboratory, anti-cardiolipin (anti-CL) (IgG/IgM isotype) by ELISA, in serum or plasma, at medium or high titers (e.g.,>40 GPL or MPL or above the 99th percentile), anti-β2 Glycoprotein-I (anti-β2GPI) (IgG/IgM isotype) by ELISA, in serum (above the 99th percentile), and lupus anticoagulant (LA) according to the guidelines of the International Society on Thrombosis and Hemostasis. Finally, C3 and C4 serum concentrations were determined by means of radial immunodiffusion.

### Disease activity and chronic damage

Disease activity was assessed by using the SLE Disease Activity Index 2000 (SLEDAI-2K). [27] According with the SLEDAI-2K values, we evaluated the number of flares and periods of persistently active disease (PAD) occurring during the follow-up. Flare was defined as an increase in SLEDAI-2K score ≥4 from the previous visit with a minimum interval of 2 months between visits; PAD as a SLEDAI-2K score ≥4, excluding serology alone, on ≥ 2 consecutive visits, with a minimum interval of 2 months between visits. [28, 29]

The Systemic Lupus International Collaborative Clinics/American College of Rheumatology (SLICC/ACR) Damage Index (SDI) was applied to evaluate the chronic damage. [30]

### Neurocognitive assessment

All patients underwent a comprehensive cognitive-behavioral neuropsychological assessment, performed by the same neurologist (CM) at baseline and after 10 years of follow-up.

Neurocognitive assessment was performed during a 1-hour interview and included standardized testing for five domains: memory, attention, abstract reasoning, executive and visuospatial functions. This assessment included those tests from the ACR and the CSI standardized in an Italian population, and was specifically designed to detect the fronto-subcortical dysfunction typical of SLE.

A Minnesota Multiphasic Personality Inventory (MMPI) was administered to all the patients in order to exclude the influence of behavioral abnormalities on cognitive dysfunction. [31] The following tests were used:

- Mini Mental State Examination (MMSE) for general cognitive status [32, 33, 34].
- Rey Auditory Verbal Learning Test and Digit Span forward, two efficient neuropsychological instruments for testing verbal memory;
- Immediate Visual Memory Test (an Italian visuospatial test) and Corsi Block-Tapping Test forward, used to measure visuospatial memory;
- Copying of Drawings with and without elements of programming, two common tools to evaluate visuospatial abilities;
- Attentive Matrices for both selective and sustained attention;
- Raven’s Progressive Matrices, a widely used non-verbal intelligence test for abstract reasoning;
- Digit Span backward, Corsi Block-Tapping Test backward, Phonological Verbal Fluency Test, Trail Making Test A, Trail Making Test B, Wisconsin Card Sorting Test, Analogies Test and Time and Weight Estimation Test, STEP, to investigate deeply the presence of executive dysfunctions. [15]

Unadjusted analysis was performed as previously described. [15, 35]

Briefly, for each patient, the raw scores from each test were compared with published norms (age-, sex-, and education level-corrected, when necessary) and transformed into Z scores to express the deviation from the normal mean [Z = (raw data2test mean)/test standard deviation]. Mean domain Z scores (MDZs) were defined as the average of the Z scores from the tests comprising each domain. To indicate cognitive function as a composite score, the Z score for each domain was transformed into a Domain Cognitive Dysfunction score (DCDs), with higher values representing more impairment in a particular domain. The sum of all DCDs across the five domains resulted in the Global Cognitive Dysfunction score (GCDs), which was transformed into a Global Cognitive Dysfunction category (GCDc).This method was summarized in Table 1.

**Table 1:** Scoring and categorization of cognitive dysfunction*.

**Test raw scores**	Obtained from performance on the neurocognitive testing
**Test Z scores**	Compared with age- and sex-matched published normal values
**Mean Domain Z scores (MDZs)**	Average of the Z scores in the tests comprising each domain
**Domain Cognitive Dysfunction Score (DCDs)**	1) if MDZs≥ -1, then DCDs = 0; 2) if -2≤MDZs<- 1, then DCDs = 1; 3) if MDZs <-2, then DCDs = 2;
**Global Cognitive Dysfunction Score**	Sum of Domain Cognitive Dysfunction Scores over the 5 domains (max 10)
**Global Cognitive Category**	Defined from Global Cognitive Dysfunction Score (GCDs)
**Absent**	GCDs 0–1
**Mild**	GCDs 2–3
**Moderate**	GCDs 4–5
**Severe**	GCDs ≥6

* The composite score is constructed from the bottom to the top of the table.

### Statistical analysis

The statistical calculations were performed using Statistical Package for Social Sciences 13.0 (SPSS, Chicago, IL, USA) and GraphPad 5.0 (La Jolla, CA, USA). Normally distributed variables were summarized using the mean±SD, and non-normally distributed variables by the median and interquartile range. Wilcoxon’s matched pairs test and paired t-test were performed. Univariate comparisons between nominal variables were calculated using chi-square (x^2^) test or Fisher’s exact test where appropriate. Two-tailed P values were reported, P values less than or equal to 0.05 were considered significant. GCDs were compared in patients grouped by antibody level. The binary outcomes variable for the antibody testing were serum autoantibody status, defined either as present versus absent or low/absent versus high. The results were verified through analysis of the domain Z scores and single-test Z scores. Descriptive statistics were computed for all study variables. Multivariable logistic regression analysis was performed including only variables that achieved P value <0.100 in the univariate analysis were included for calculation.

## Results

After a mean follow-up of 119.4±7.2 months, 43 SLE patients (74.1%; M/F 5/38) were re-evaluated: at baseline, these patients showed a mean age of 36.7±10.0 years (range 19–58 years), a mean disease duration of 110.9±73.6 months, a mean ±SD duration of scholar education of 12.2±3.5 years.

Considering the 15 SLE patients lost to follow-up, 13 refused to participate to the second evaluation, and two patients died (one for complicated infection and one for cardiovascular event). No significant differences between re-evaluated and missing patients were observed as regards demographic, clinical and laboratory features. In Table 2, we reported the main characteristics observed in the 43 SLE patients. These manifestations were cumulative and referred to the disease history.

**Table 2:** Clinical and laboratory features of SLE patients (N = 43) enrolled in the study.

**ACR Criteria (N/%)**	**T0**	**T1**	P
Malar Rash	30/69.8	30/69.8	NS
Discoid Lupus	5/11.6	9/20.9	NS
Photosensitivity	17/39.5	19/44.2	NS
Mucosal Ulcers	16/37.2	16/37.2	NS
Arthritis	39/90.7	39/90.7	NS
Serositis	13/30.2	17/39.5	NS
Kidney involvement	16//37.2	17/39.5	NS
Neurologic manifestations	33/76.7	37/86.0	NS
Seizure	6/13.9	6 /13.9	
Vascular disease	6/16.3	7/16.3	
Mood disorders	29/67.4	29/67.4	
Headache	22/51.2	22/51.2	
Neuropathy	1/ 2.3	6/13.9	
Myelopathy	0	2/4.6	
Psychosis	7/16.3	7/16.3	
Myasthenia gravis	0	1 /2.3	
Movement disorders	1/2.3	1 /2.3	
Haematological disorders	36/83.7	36/83.7	NS
Immunologic features	33/76.7	38/88.4	NS
ANA positivity	43/100	43/100	NS
**Autoantibodies (N/%)**			
	**T0**	**T1**	
Anti-dsDNA	25/83.3	30/69.8	NS
Anti-phospholipid	25/83.3	29/67.4	NS
aCL	18/41.8	23/53.5	NS
anti-B2GPI	11/25.6	13/30.2	NS
LA	9/20.9	11/25.6	NS

**Legend:** ACR: American College of Rheumatology; ANA: antinuclear antibodies; B2GPI: Beta2 Glycoprotein-I; LA: Lupus Anticoagulant.

With regard to other autoimmune diseases, eleven patients (25.6%) were affected by anti-phospholipid syndrome (APS), six (13.9%) by Sjögren’s Syndrome. Furthermore, the presence of comorbidity was registered: arterial hypertension was identified in 21 patients (48.8%), thyroid pathology in 15 (34.9%), dyslipidemia, defined as raised plasma triglycerides (≥ 150 mg/dl) and/or low HDL-C (<40 mg/dl in men and <50 mg/dl in women) [36], in 11 (25.6%), diabetes in three (7.0%). During the follow-up, five patients (11.6%) developed peripheral neuropathy, 3 (7.0%) a cerebrovascular event, and 2 (4.6%) transverse myelitis. Finally, one patient (2.3%) developed *myasthenia gravis*. In Table 3 the treatments at baseline (T0) and after 10 years (T1) were reported. A significant reduction in mean weekly glucocorticoid (GC) dosage was identified (P = 0.006).

**Table 3:** Treatments at baseline (T0) and after 10 years (T1) of 43 SLE patients.

	T0	T1	P
	(N = 43)	(N = 43)	
**GC treatment, N/%**	31 (72.1)	33 (76.7)	NS
Mean dosage±SD (mg/weekly)	60.6± 63.0	31.3± 36.9	0.006
**Hydroxychloroquine, N/%**	20 (46.5)	21 (48.8)	NS
**Immunosuppressant drugs (N/%)**			
Methotrexate	2 (4.6)	1 (2.3)	NS
Azathioprine	2 (4.6)	8 (16.6)	0.004
Cyclosporine A	6 (13.9)	3 (7.0)	NS
Mycophenolate Mofetil	3 (7.0)	7 (16.3)	0.04
Cyclophosphamide	2 (4.6)	-	NS
Rituximab	-	2 (4.6)	NS
Leflunomide	1 (2.3)	-	NS
**Anti-thrombotic treatment (N/%)**			
Low dose aspirin	12 (27.9)	10 (23.2)	NS
Anticoagulation	4 (9.3)	5 (11.6)	NS

Data concerning the disease activity and chronic damage are reported in Table 4. A significant increase of chronic damage, assessed by using SDI, was observed after 10 years (P = 0.001).

**Table 4:** Disease activity and chronic damage at baseline (T0) and after 10 years (T1) in 43 SLE patients.

Features	T0	T1	P
SLEDAI-2K (mean±SD)	2.9±4.4	3.8±3.9	NS
SDI (mean±SD)	1.6±1.8	3.1±2.6	0.001
Number of Flares (mean±SD)	-	2.1±2.1	-
PAD: Number (mean±SD)	-	1.1±1.1	-
PAD: Duration (mean±SD)	-	18.2±13.9	-

### Cognitive assessment

No alterations in the MMSE were identified in SLE patients at T0 and T1, excluding a severe impairment of global cognitive status. The assessment by using MMPI depression scale confirmed the presence of this mood disorder in 29 patients, previously diagnosed by a neuropsychiatrist specialist. With regard to GCDs, CI was identified in 20.9% (9 patients) at T0 and in 13.9% (6 patients) at T1 (P = NS). This impairment was prevalently mild at T0 (55.5%) and mild or moderate at T1 (36.3% for both degrees).

Considering patients with CI at baseline, 55.5% experienced an improvement, while the other patients remained stable. Only one patient among the 34 without CI at baseline experienced a neurocognitive dysfunction with mild impairment.

**Fig 1:**
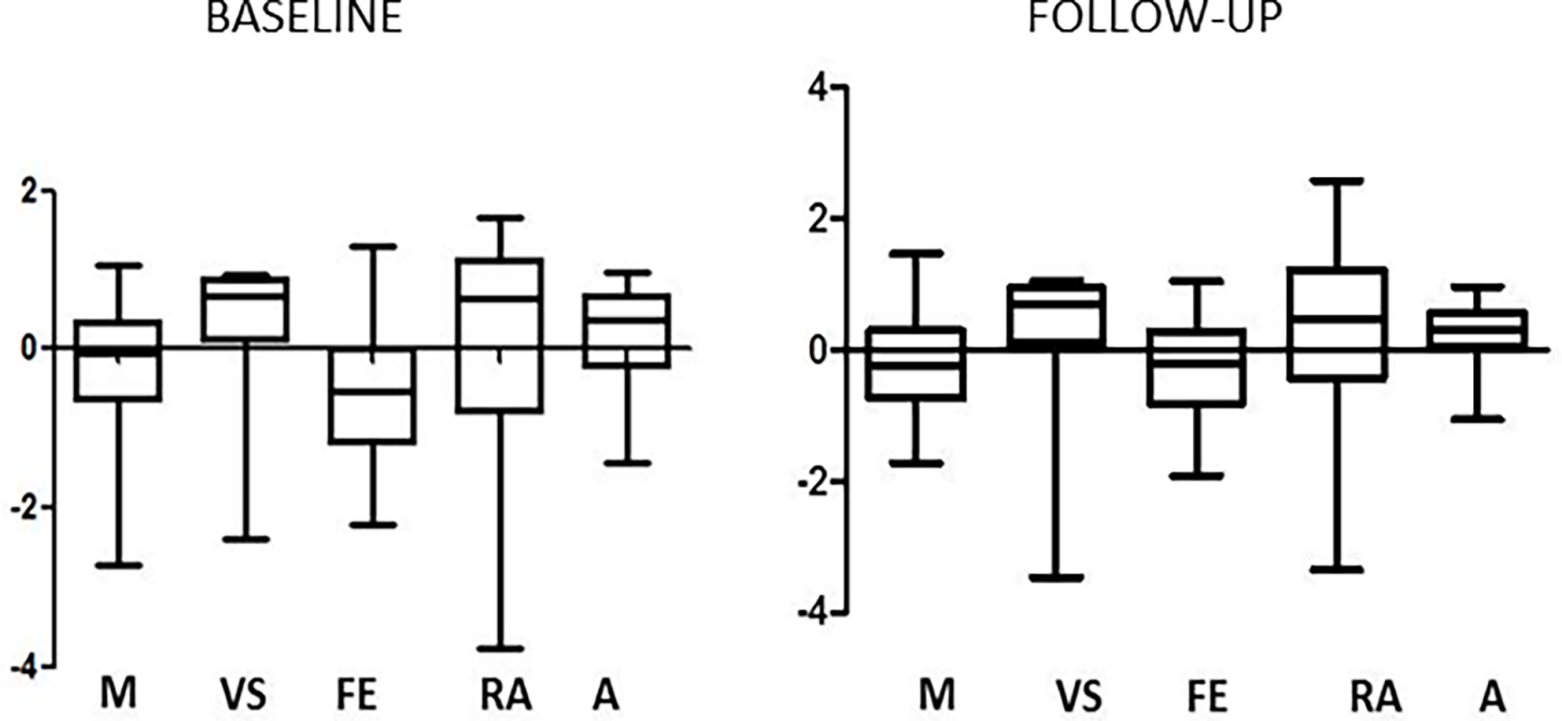
Distribution of neurocognitive impairment, expressed as MDZ scores ±SD, in the patients enrolled at baseline (T0) and after 10 years.

The mean domain Z scores were graphically represented in Fig 1: the domain referring to executive functions was the most compromised at baseline (0.5±0.8) and after 10-year follow-up (-0.3±0.7).

**Fig 2:**
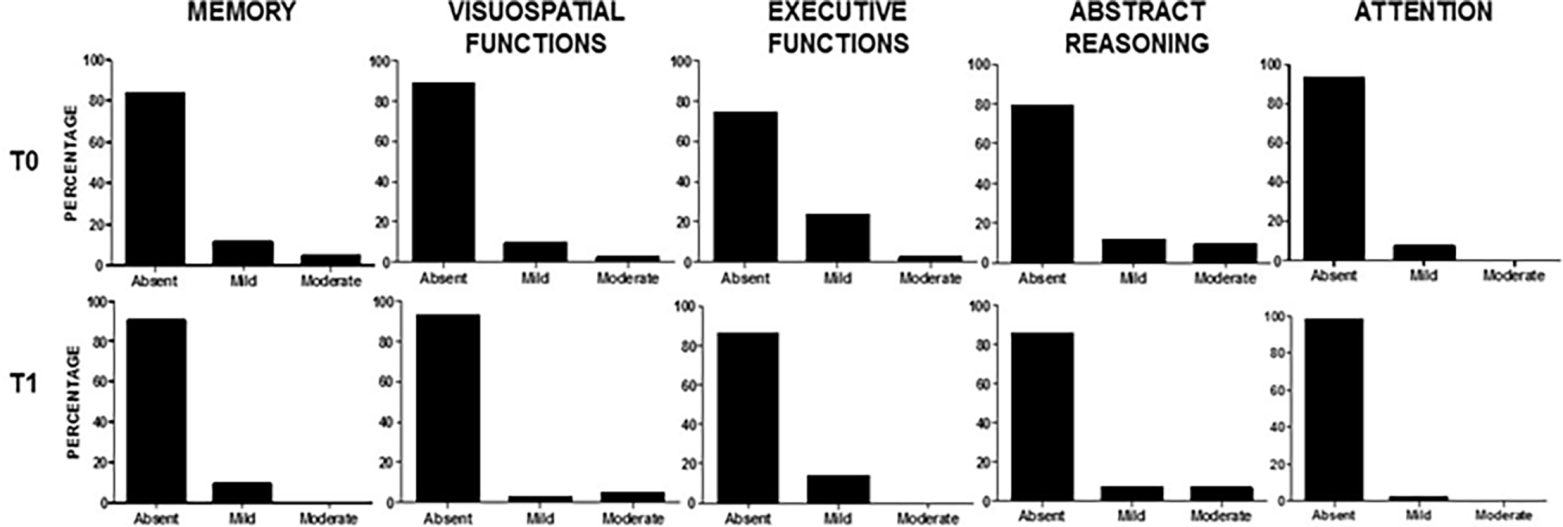
Percentage of patients with neurocognitive impairment, expressed as DCDs, in all the domains evaluated at T0 and T1. Absent: DCD = 0; Mild: DCD = 1; Moderate: DCD = 2.

All the domains showed an improvement over-time, with a significant difference from baseline for executive function (P = 0.04). After transforming the MDZs into DCDs, the percentage of patients with impairment in the different domains was calculated as shown in Fig 2.

The domain referring to executive functions was the most frequently involved at baseline (mild/moderate DCDs: 11 patients, 25.6%); this frequency was significantly reduced at the follow-up, when the domain was compromised in six patients (13.9%, P = 0.04). Of note, after 10 years the frequency of impairment was reduced in all the domains evaluated even if without reaching significant difference.

Moreover, the presence of dyslipidemia, considered in our statistical analysis as a categorical variable, resulted significantly associated with memory impairment (P = 0.02), executive functions (P = 0.02) and abstract reasoning (P = 0.03) at the baseline, and with visuospatial functions (P = 0.009) and abstract reasoning (P = 0.004) at T1. Of note, worsening of visuospatial functions was significantly associated with dyslipidemia and positivity for LA (P = 0.04 for both parameters). Finally, the GCDs significantly correlated with SDI value at the baseline (r = 0.3; P = 0.04) and at T1 (r = 0.3; P = 0.03).

No correlations between CI and demographical characteristics (including age and education level), SLE clinical features, ongoing and past treatments (specifically GC, immunosuppressants and antithrombotic drugs), comorbidities, activity and damage indices were observed in the present SLE cohort at baseline and follow-up.

Moreover, all the lupus features (such as disease manifestations, antibody levels, disease and damage scores) and ongoing and past medications (including DMARDS, anticoagulants and glucocorticoids’ dosage) were considered in the multivariate logistic regression analysis, without identifying significant association.

Moreover no significant associations were identified between CI and behavioral abnormalities evaluated by MMPI scales.

## Discussion

In the present longitudinal study, the CI changes after 10 years of follow-up were evaluated in a cohort of Caucasian SLE patients. We observed an improvement of this manifestation in the majority of patients evaluated, with a worsening in only 10% of subjects. Furthermore, all cognitive functions improved and a statistically significant difference was achieved for the executive functions.

Interestingly, the same operator performed the neurocognitive assessment at baseline and after 10 years, safeguarding the reliability of the results and reducing the risk of performance bias. At the same time, the patients were followed constantly in our Lupus Clinic during the 10 years follow-up, suggesting that clinical decisions among this time lapse were homogenous.

Despite the high number of studies evaluating SLE-related CI, few data have analyzed its over-time changes, with a maximum follow-up of 5 years. To sum up, in most of the studies a fluctuating or stable trend was identified more frequently than worsening. [22–25]

The one-year follow-up study of Carlomagno and colleagues demonstrated a stable trend in more than 90% of SLE-patients. [23]The 5-year evaluation of a 70-patients SLE cohort conducted by Hanly et al. in 1997 identified a reduction of CI prevalence from 21% to 13%. Moreover, the same study suggested that the presence of previous neuropsychiatric events could predict a CI worsening. [22] According with these results, Gao and colleagues reported a worsening of cognitive ability after 12-month follow-up only in NPSLE patients, in comparison with non-NPSLE and healthy control. [25] Waterloo and colleagues investigated the changes of different neuro-psychological variables after 5years of follow-up in a 28-patients cohort. In particular, a stable pattern was identified in all variables but two (namely, Category test and Seashore Rhythm Test). [24]

We previously evaluated a 58-patients SLE cohort in order to assess the CI prevalence and the possible association with other clinical and laboratory SLE-related features. We observed a mild GCDs impairment in 19% of patients, moderate (GCDs 4–5) in 7% and severe in 5%.(15) In this cohort, the impairment of visuospatial domain resulted the most compromised and significantly associated with aCL IgM levels. Moreover, disease activity and chronic damage correlated with different domains. [15]

In the present study, we re-evaluated 43 out of 58 patients (74.1%), observing a reduction of the CI prevalence from 20.9to 13.9% after 10-yearfollow-up. Specifically, half of patients with CI at the baseline showed an improvement and only 10% a worsening. These results reinforce those obtained in above-described studies: in our cohort, even the majority of patients experienced an improvement of cognitive functions, differently from the stable trend previously described. We could hypothesize that the improved management of SLE patients and new therapeutic strategies allowed these results. In fact, previous studies were conducted more than 10 years ago and a wider knowledge on the SLE management as well as of therapeutic arrows can be expected in this time interval. [37, 38]

This suggestion was confirmed indirectly by the comparison of treatments at baseline and after 10 years. Of note, a significantly lower mean weekly dosage of GC was documented at the follow-up, with a significant increase of percentage of patients assuming dosage lower than 35mg/weekly of prednisone equivalents. Moreover, in our cohort we observed a significant increase of immunosuppressant drugs administration (in particular, Azathioprine and Mycophenolate) and the appearance of biological drugs usage. Of note, in the last years a growing use of Mycophenolate was registered in Lupus cohorts, demonstrating the efficacy of this drug in other than renal involvement features, such as neurological manifestations. [39]

Furthermore, all patients were treated by antimalarial drugs and/or immunosuppressant and, in case of positivity for aPL antibodies, by anti-thrombotic therapy. Taken together, these treatments could influence the different inflammatory and thrombotic pathogenic mechanisms potentially determining CI. Nonetheless, it should be considered that the low mean age at the baseline (lower than 40 years) and the high scholar level of our cohort could influence the ability to perform neurocognitive assessment.

Furthermore, we evaluated the association between CI and the different clinical/laboratory SLE-related features, as well as cardiovascular comorbidities. We observed a significant association between dyslipidemia and CI in all the domains, except for the attention, at baseline and after 10 years. Moreover, this comorbidity significantly correlated with the worsening of visuospatial functions. These results are in agreement with previous evidences: in particular, a multicenter Italian study including about 1.000 SLE patients identified dyslipidemia as a risk factor for the presence of CI. [40]

We could hypothesize, as a possible pathogenetic explanation, a concurrent subclinical vascular injury in both dyslipidemic and cognitive impaired patients which may justify this association.

In addition, our analysis confirms the possible pathogenic role of aPL in the CI development: a significant correlation between LA positivity and the worsening in visuospatial functions was identified in our cohort. This finding suggests that aPL may act determining not only a focal damage (at level of thrombotic event), but also a more diffuse damage, with a direct mechanism on neural cells. [41, 42] Moreover, disease activity and chronic damage seem to influence CI. Interestingly, the presence of flares assessed according with SLEDAI-2K modifications, resulted significantly associated with a worsening in the memory domain. This result suggests that the prevention of disease relapse could control the involvement of cognitive functions.

We would highlight that generally MMPI is not an optimal battery to evaluate mood disorders. However, since at baseline evaluation, performed about 10 years ago, MMPI has been chosen to exclude the influence of behavioral abnormalities on cognitive dysfunction, we decided to keep the same protocol in order to maintain continuity and consistency of our longitudinal study. Nonetheless we observed that the assessment by MMPI depression scale indeed confirmed the presence of this mood disorder in the same patients previously diagnosed by a neuropsychiatrist specialist. Thus, all MMPI scales were included in the multivariate analysis but did not correlate with CI identified.

In conclusion, the present study provides data concerning the changes over-time of SLE-related CI, by considering for the first time a follow-up of 10 years. Our data demonstrated a stability of cognitive functions, with a trend to the improvement in all the evaluated domains. The risk factors for a worse prognosis resulted the positivity for aPL, in particular LA, and the presence of a concomitant dyslipidemia. Moreover, the prevention of disease relapse and chronic damage development is mandatory in order to prevent CI worsening.
